# Pain, pain intensity and pain disability in high school students are differently associated with physical activity, screening hours and sleep

**DOI:** 10.1186/s12891-017-1557-6

**Published:** 2017-05-16

**Authors:** Anabela G. Silva, Pedro Sa-Couto, Alexandra Queirós, Maritza Neto, Nelson P. Rocha

**Affiliations:** 1 School of Health Sciences, University of Aveiro, Campus Universitário de Santiago, 3810-193 Aveiro, Portugal; 2 Center for Health Technology and Services Research (CINTESIS), Piso 2, edifício nascente, Rua Dr. Plácido da Costa, s/n, 4200-450 Porto, Portugal; 3 Center for Research and Development in Mathematics and Applications (CIDMA), Department of Mathematics (DMAT), University of Aveiro, Aveiro, Portugal; 4 Institute of Electronics and Telematics Engineering of Aveiro (IEETA), University of Aveiro, Campus Universitário de Santiago, 3810-193 Aveiro, Portugal; 5 Primary Healthcare Center, Av. Dr. Rocha Madail,S/N, Ílhavo, Portugal; 6 Department of Medical Sciences, University of Aveiro, Campus Universitário de Santiago, 3810-193 Aveiro, Portugal

**Keywords:** Pain, Disability evaluation, Physical activity, Screen time, Sleep

## Abstract

**Background:**

Studies exploring the association between physical activity, screen time and sleep and pain usually focus on a limited number of painful body sites. Nevertheless, pain at different body sites is likely to be of different nature. Therefore, this study aims to explore and compare the association between time spent in self-reported physical activity, in screen based activities and sleeping and i) pain presence in the last 7-days for 9 different body sites; ii) pain intensity at 9 different body sites and iii) global disability.

**Methods:**

Nine hundred sixty nine students completed a questionnaire on pain, time spent in moderate and vigorous physical activity, screen based time watching TV/DVD, playing, using mobile phones and computers and sleeping hours. Univariate and multivariate associations between pain presence, pain intensity and disability and physical activity, screen based time and sleeping hours were investigated.

**Results:**

Pain presence: sleeping remained in the multivariable model for the neck, mid back, wrists, knees and ankles/feet (OR 1.17 to 2.11); moderate physical activity remained in the multivariate model for the neck, shoulders, wrists, hips and ankles/feet (OR 1.06 to 1.08); vigorous physical activity remained in the multivariate model for mid back, knees and ankles/feet (OR 1.05 to 1.09) and screen time remained in the multivariate model for the low back (OR = 2.34. Pain intensity: screen time and moderate physical activity remained in the multivariable model for pain intensity at the neck, mid back, low back, shoulder, knees and ankles/feet (Rp^2^ 0.02 to 0.04) and at the wrists (Rp^2^ = 0.04), respectively. Disability showed no association with sleeping, screen time or physical activity.

**Conclusions:**

This study suggests both similarities and differences in the patterns of association between time spent in physical activity, sleeping and in screen based activities and pain presence at 8 different body sites. In addition, they also suggest that the factors associated with the presence of pain, pain intensity and pain associated disability are different.

## Background

Musculoskeletal pain not associated with a disease is very common in childhood and adolescence reaching a lifetime prevalence as high as 40% [1]. The body regions more commonly affected are the neck/shoulder, the low back and the lower limbs [2] and pain is usually reported in multiple body sites: 20 to 40% of girls and 8 to 23% of boys report pain in at least 3 body regions [2, 3]. Furthermore, pain negatively impacts day-to-day activities such as sleeping, school activities, leisure activities or meeting with friends [4].

Several factors have been associated with the reporting of pain across the age range of 11 to 19 years old. These included physical activity (PA), screen time and sleep. Nevertheless studies show contrasting results. For example, PA has been shown to be associated with an increased probability of reporting pain [5], to have a protective effect in relation to pain [6] or to have no association with pain [7]. The strength of the association between screen time and pain has been shown to vary across studies and type of screen based activity [6, 8, 9]. Differences regarding the body region investigated and whether a single predictor or multiple predictors are considered in the analysis may help explain discrepancies between studies. Different body regions seem to be associated with pain of different nature: while pain in the neck is predominantly idiopathic, pain related to trauma is more common in the lower limb than in the neck pain [10]. Traumatic and non-traumatic pain (defined as including all pains not associated with direct trauma), have different risk factors and a different impact on daily activities [11]. Furthermore, while physical activity is a risk factor for trauma [12], it is also associated with a number of physical and psychological benefits [13, 14]. Conceivably, it could be associated with pain in a body region more prone to pain of traumatic origin and have a protective effect on a body region where pain is more of a non-traumatic nature. Furthermore, evidence suggests that PA, screen time and sleep correlate with each other: practicing more PA seems to be associated with better sleep [15] and higher screening time is associated with less sleep quantity [16]. Furthermore, it has already been shown that sleep partly mediates the association between computer use and somatic symptoms [17]. Thus, it is possible that PA, screen time and sleep variables could act as confounders of each other in the association with pain. Recall bias may also have an impact on study results as most studies report on chronic pain and rely on the adolescent’s ability to recall pain over the last 3 months. Nevertheless, pain memory for long intervals of time may be inaccurate [18].

We hypothesized that the association between pain and PA, screen time and sleep will vary depending on the painful body site considered and on the type of association investigated (univariate or multivariate). This study aims to explore and compare the association between time spent in self-reported PA, in screen based activities and sleeping and i) pain reporting in the last 7-days for 9 different body sites; ii) pain intensity at 9 different body sites and iii) global pain associated disability.

## Methods

### Ethics, consent and permissions

Ethical approval was obtained from the Council of Ethics and Deontology, Faculty of Medicine, University of Porto. Students had to provide their written informed consent and for students aged 16 or younger both students and their legal guardian provided their written informed consent.

### Study design and population

This is a cross sectional study that took place in the 5 schools of the Council of Ílhavo with the 7th or higher grades (age range of students: 13 to 19 years old). A total of 1330 students were enrolled at the time of data collection and all were invited to enter the study. Data collection took place between March and June 2014 and was performed through an online questionnaire purposefully developed for this study. Students were given an individual login and password and filled the questionnaire during a physical education lesson. Students missing school on the day of data collection were invited to complete the questionnaire on another day.

### Measures

#### Demographic data

Students were asked to enter data on sex, age, school year, height and weight.

### Pain

A previously adapted version of the Nordic Musculoskeletal Questionnaire (NPQ) was used in this study. Students were asked if they felt pain during the past 7 days in the neck, shoulders, elbows, wrists/hands, mid back, lumbar region, hips, knees and ankles/feet. When signaling pain, students were prompted to report on its intensity for each body site using a numeric pain rating scale from 0 (no pain) to 10 (worst pain imaginable). The body chart of the NPQ was included in the questionnaire to guide students in their answers. When analyzing the data, a variable number of pain sites was created by simply counting the number of body sites (out of the 9 body sites) with pain.

### Disability

In order to assess perceived disability we used an index previously reported by Hoftun et al. [2]. Participants were asked to indicate if they felt that the following statements applied to them: (1) I have difficulties falling asleep because of pain and/or pain disturbs my sleep; (2) because of pain I have difficulties sitting during a lesson; (3) pain disturbs me if I walk more than 1 km and; (4) pain disturbs me during physical exercise class; (5) pain disturbs me during my leisure activities. Each statement that applied counted as one point and a total score for each participant was calculated to a maximum of 5 points (one point for each verified statement).

### Physical activity

Students were asked whether: they participated in moderate physical activities (activities that might increase heart beating and breathing slightly) and/or in vigorous physical activities (activities that make the heart beat harder and the breathing noticeably faster) other than the physical education classes. Those answering yes had to provide information on: i) type of activity, ii) number of days per week (1 to 7) and iii) mean duration of each activity per session (minimum 10 min). These questions were adapted from Lang et al. [19], piloted using 6 students aged 13 to 18 years old, who were probed on their understanding of questions, and reviewed by a physical education teacher. Response options for questions related to physical activity were based on published guidelines [20–22] and were: i) walking to school, ii) walking in general (other than to school), iii) cycling, iv) skating, v) roller skating, or vi) others, for the moderate physical activities question; and i) basket, ii) running, iii) martial arts, iv) handball, v) ballet, vi) football, vii) rowing, viii) volleyball, ix) swimming, x) gymnastics or xi) other, for the vigorous physical activities question. In order to calculate the total time of moderate and vigorous PA per week for each student, the daily amount of time spent in each activity was multiplied by the number of days that the activity was performed and, then added to the weekly time spent in other activities, if the student reported more than one activity.

### Time spent in screen based activities

Time spent in screen based activities was assessed using 4 closed questions on the number of hours spent each day:

1. Watching TV/DVDs: this includes watching TV programs and videos;
2. Playing: this includes using TV, computers, or PlayStation to play wired or standalone games;
3. Using mobile phones: this includes using phones to play or to communicate;
4. Using computers: this includes desktop, portable computers or tablets, both to communicate or to manage information.

Each question had 5 possible response options: (1) do not use; (2) use 1 h or less per day; (3) use 2 to 3 h per day; (4) use 4 to 5 h per day; and (5) use more than 5 h per day. Questions on time spent in screen based activities were adapted from Hakala et al.[8], who also assessed its test-retest reliability and reported K values between 0.45 and 0.65, suggestive of fair to good agreement.

### Sleep

Sleeping hours were assessed with a closed question (On average, how many hours per day do you sleep?) with the following response options: i) less than 6 h; ii) 6 to 7 h; iii) 8 to 9 h; iv) 10 h or more. This was based on the National Sleep Foundation guidelines that teenagers should get between 8 to 10 h sleep.

### Statistical analysis

Summary statistics were reported as means and standard deviations for continuous variables and as counts and percentages for categorical variables. Potential predictors factors (Odds Ratios, OR, and 95% Confidence Intervals, CI) associated to different pain sites were explored in univariable and multivariable analysis (only for the variables presenting p ≤ 0.10 in univariable model) performed using binary logistic regression models. Multiple linear regression models were used to predict pain intensity at different body sites and the overall score for disability. All the multivariable regression models were performed using a forced entry method (all the considered variables are entered into the equation in one step). The independent variables used were the same for all prediction models (gender, age, body mass index (BMI), sleeping hours, time spent in moderate PA, time spent in vigorous PA, watching TV/DVDs, playing, using mobile phones and using computers). Additionally, in the model for perceived disability, the independent variables “number of pain sites” and “pain intensity” were also considered while for the prediction of pain intensity only the former was taken into account. The assumptions for the regression models (Hosmer and Lemeshow test for logistic regression and normality of the residuals for linear regression) were verified. All statistical analyses were performed using SPSS® Software, version 22.0 (SPSS, Inc., Chicago, IL) and *p*-values under 0.05 were considered significant.

## Results

A total of 969 (72.9%) students aged (mean ± SD) 15.6 ± 1.8 years answered the questionnaire. A total of 652 (67.3%) students reported pain in the last 7 days in at least one body segment. The knees (22.6%), the low back (19.9%), the shoulders (17.3%) and the neck (17.1%) were the body sites where pain prevalence was higher. Mean (±SD) pain intensity per body site varied between 3.6 ± 1.8 (in the neck) and 4.4 ± 2.3 (knees). Of the 652 students who reported pain in at least one body site, 371 (56.9%) reported difficulties performing at least one activity due to pain. A detailed description of the sample is presented in Table 1 and a detailed description of pain prevalence per body region, sex and age is presented in Table 2. We excluded the elbow from the regression analysis as only 30 participants reported pain in this body site.

**Table 1:** Sample characterization.

		N (%)
Gender (*n* = 969)	Male	467(48.2)
	Female	502(51.8)
Age (years) (*n* = 969)	13–15	511(52.7)
	≥16	458(47.3)
Weight (Kg) (*n* = 969)		57.3 ± 11.9
Height (m) (*n* = 969)		1.64 ± 0.11
Scholar level (*n* = 969)	7° grade	212 (21.9)
	8° grade	192 (19.8)
	9° grade	180 (18.6)
	10° grade	101 (10.4)
	11° grade	141 (14.6)
	12° grade	143 (14.8)
Sleeping hours (*n* = 969)	≤6 h	22 (2.3)
	[6;7] hours	322 (33.2)
	[8;9] hours	576 (59.4)
	≥10 h	49 (5.1)
Disability (*n* = 652)^a^	No disability	281 (43.1)
	Difficulty with 1 activity	241 (37.0)
	Difficulty with 2 activities	81 (12.4)
	Difficulty with 3 activities	35 (5.4)
	Difficulty with 4 activities	14 (2.1)
	Difficulty with 5 activities	0 (0)
Disability index ([1;5]; *n* = 371)	Mean ± SD	1.5 ± 0.8
Number of pain sites (*n* = 652)	Pain in 1 body site	284 (29.3)
	Pain in 2 body sites	190 (19.6)
	Pain in 3 body sites	90 (9.3)
	Pain in ≥ 4 body sites	88 (9.1)
Number of pain sites ([1;9], *n* = 652)^a^	Mean ± SD	2.1 ± 1.3
Physical activity (*n* = 968)	Moderate (Yes)	714 (73.7)
	Vigorous (Yes)	575 (59.3)
Watching TV/DVDs (*n* = 969)	No	52 (5.4)
	≤1 h	404 (42.0)
	[2;3] hours	399 (41.5)
	≥4 h	107 (11.1)
Playing (*n* = 969)	No	105 (10.9)
	≤1 h	345 (35.9)
	[2;3] hours	313 (32.5)
	≥4 h	199 (20.7)
Using mobile phones (*n* = 969)	No	113 (11.7)
	≤1 h	361 (37.5)
	[2;3] hours	195 (20.3)
	[4;5] hours	105 (10.9)
	≥5 h	188 (19.5)
Using computers (*n* = 969)	No	86 (8.9)
	≤1 h	375 (39.0)
	[2;3] hours	311 (32.3)
	≥4 h	190 (19.8)

^a^652 was the number of participants reporting pain in at least one body site

**Table 2:** Pain presence and pain intensity by gender and age [mean ± standard deviation (percentage)]

Body site		Girls			Boys			Total
		13–15	≥16	Total	13–15	≥16	Total	
At least one body site		188(51.2)	179(48.8)	367(73.1)	142(49.8)	143(50.2)	285(61.0)	652(67.3)
Neck	Pain	56(51.4)	53(48.6)	109(21.7)	28(49.1)	29(50.9)	57(12.2)	166(17.1)
	Intensity	3.8 ± 1.9	3.9 ± 1.7	3.9 ± 1.8**	3.2 ± 1.8	3.0 ± 1.4	3.1 ± 1.6**	3.6 ± 1.8
Shoulder	Pain	52(47.3)	58(52.7)	110(21.9)	25(43.1)	33(56.9)	58(12.4)	168(17.3)
	Intensity	4.2 ± 2.0	4.3 ± 2.0	4.3 ± 2.0	4.2 ± 1.6	3.8 ± 1.5	4.0 ± 1.5	4.1 ± 1.8
Elbow	Pain	8(53.3)	7(46.7)	15(3.0)	10(66.7)	5(33.3)	15(3.2)	30(3.1)
	Intensity	4.4 ± 2.4	5.3 ± 1.6	4.8 ± 2.1	3.2 ± 2.0	3.2 ± 3.0	3.2 ± 2.2	4.0 ± 2.3
Wrist/hand	Pain	50(58.8)	35(41.2)	85(16.9)	24(42.9)	32(57.1)	56(12.0)	141(14.6)
	Intensity	4.4 ± 2.0*	3.7 ± 2.2*	4.2 ± 2.1	3.1 ± 1.7*	4.0 ± 2.1*	3.6 ± 1.9	3.9 ± 2.1
Mid back	Pain	39(48.8)	41(51.3)	80(15.9)	24(50.0)	24(50.0)	48(10.3)	128(13.2)
	Intensity	4.8 ± 2.3	3.4 ± 1.5	4.1 ± 2.0	4.3 ± 2.1	3.5 ± 1.7	3.9 ± 1.9	4.0 ± 2.0
Low back	Pain	62(50.0)	62(50.0)	124(24.7)	32(46.4)	37(53.6)	69(14.6)	193(19.9)
	Intensity	4.4 ± 2.1	4.7 ± 2.1	4.6 ± 2.1*	4.1 ± 2.1	3.7 ± 1.9	3.9 ± 2.0*	4.3 ± 2.1
Hip	Pain	33(53.2)	29(46.8)	62(12.4)	23(45.1)	28(54.9)	51(10.9)	113(11.7)
	Intensity	4.3 ± 2.0	4.6 ± 1.8	4.4 ± 1.9***	3.4 ± 1.7	2.9 ± 1.1	3.1 ± 1.4***	3.8 ± 1.8
Knee	Pain	67(52.3)	61(47.7)	128(25.5)	56(61.5)	35(38.5)	91(19.5)	219(22.6)
	Intensity	4.8 ± 2.5	4.5 ± 2.2	4.7 ± 2.4	4.0 ± 2.2	4.1 ± 1.9	4.0 ± 2.0	4.4 ± 2.3
Ankle/foot	Pain	45(47.9)	49(52.1)	94(18.7)	47(52.2)	43(47.8)	90(19.3)	184(19.0)
	Intensity	4.5 ± 2.5	4.2 ± 2.0	4.3 ± 2.2	3.9 ± 2.6	3.9 ± 2.2	3.9 ± 2.4	4.1 ± 2.3

*p < 0.05; **p < 0.01;***p < 0.001

### Pain and gender, age and BMI (univariable analysis)

When considering the association between pain in the last 7-days and gender, age and BMI per body region, results show that being a female significantly increased the odds of reporting pain at all body sites considered in the analysis (OR between 1.64 and 2.58, *p* < 0.05) except the ankles/feet. Being 15 years and older significantly increased the odds of reporting pain in the shoulders (OR = 1.57, *p* < 0.05) and no association was found between BMI and pain (Tables 3, 4 and 5).

**Table 3:** Odds ratios (OR) and 95% confidence intervals (Cl) for multivariate associations with pain intensity in last 7 days in the neck, mid back and low back

		Neck		Mid back		Low back	
		Univariable	Multivariable	Univariable	Multivariable	Univariable	Multivariable
		OR; IC95%	OR; IC95%	OR; IC95%	OR; IC95%	OR; IC95%	OR; IC95%
Gender	Female	2.58; [1.75;3.81]**	2.85; [1.90;4.27]**	2.25; [1.47;3.43]**	2.42; [1.52;3.86]**	2.42; [1.68;3.50]**	2.51; [1.68;3.78]**
Age	≥16	1.30; [0.89;1.89]		1.37; [0.91;2.07]		1.40; [0.98;2.01]*	1.47; [0.99;2.19]
Sleeping hours	≤7 h	1.98; [1.34;2.93]**	2.05; [1.36;3.08]**	2.16; [1.41;3.30]**	2.11; [1.34;3.32]**	1.64; [1.12;2.39]**	1.37; [0.90;2.08]
Physical activity	Moderate	1.06;[1.02;1.12]**	1.08;[1.03;1.13]**	1.03;[0.98;1.09]		1.05;[1.01;1.09]**	1.04;[0.99;1.09]
	Vigorous	1.02;[0.98;1.06]		1.05;[1.01;1.09]**	1.06;[1.02;1.11]**	1.03;[0.99;1.07]*	1.03;[0.99;1.08]
Using mobile phones	≤1 h	1.19;[0.62;2.29]		1.08;[0.52;2.24]	0.73;[0.34;1.57]	1.25;[0.66;2.36]	0.75;[0.37;1.52]
	[2;3] hours	1.20;[0.59;2.42]		1.07;[0.48;2.36]	0.73;[0.32;1.67]	1.00;[0.50;2.03]	0.55;[0.25;1.19]
	[4;5] hours	1.83;[0.82;4.08]		1.89;[0.79;4.54]	1.06;[0.41;2.69]	1.65;[0.74;3.65]	0.86;[0.35;2.10]
	≥5 h	1.92;[0.91;4.07]		2.74;[1.23;6.07]**	1.38;[0.58;3.26]	3.20;[1.58;6.45]**	1.45;[0.64;3.25]
Using computers	≤1 h	1.59;[0.74;3.44]		0.97;[0.46;2.03]		2.34;[1.06;5.14]**	2.43;[1.03;5.75]**
	[2;3] hours	2.13;[0.98;4.61]		1.20;[0.57;2.55]		2.33;[1.05;5.19]**	2.43;[1.04;6.01]**
	≥4 h	2.16;[0.95;4.93]		1.54;[0.70;3.42]		3.02;[1.31;6.97]**	2.14;[0.86;5.58]

*p < 0.1; ** *p* < 0.05; Only significant associations are shown; reference categories for OR: gender (male), age (13–15 years old), sleeping (≥8 h), using mobile phone (no use), using computers (no use)

**Table 4:** Odds ratios (OR) and 95% confidence intervals (Cl) for multivariate associations with pain in last 7 days in the upper limb

		Shoulders		Wrists	
		Univariable	Multivariable	Univariable	Multivariable
		OR; IC95%	OR; IC95%	OR; IC95%	OR; IC95%
Gender	Female	2.56; [1.73;3.77]**	2.97; [1.94;4.54]**	2.05; [1.37;3.07]**	2.13; [1.38;3.27]**
Age	≥16	1.57; [1.08;2.29]**	1.77; [1.17;2.69]**	1.21; [0.81;1.80]	
Sleeping hours	≤7 h	1.48; [0.99;2.10]*	1.32; [0.85;2.06]	1.87; [1.24;2.83]**	1.79; [1.15;2.77]**
Physical activity	Moderate	1.06;[1.01;1.10]**	1.06;[1.01;1.11]**	1.09;[1.03;1.14]**	1.08;[1.03;1.14]**
	Vigorous	1.04;[1.01;1.09]**	1.00;[1.00;1.01]	1.01;[0.97;1.06]	
Using mobile phones	≤1 h	0.91;[0.49;1.69]	0.66;[0.34;1.27]	1.36;[0.66;2.79]	1.14;[0.54;2.41]
	[2;3] hours	0.69;[0.35;1.39]	0.47;[0.22;0.99]	1.29;[0.59;2.80]	0.97;[0.43;2.19]
	[4;5] hours	1.47;[0.68;3.16]	0.76;[0.33;1.77]	1.89;[0.79;4.54]	1.31;[0.52;3.30]
	≥5 h	2.00;[1.00;4.01]*	1.11;[0.52;2.35]	2.56;[1.15;5.72]**	1.57;[0.67;3.68]

*p < 0.1; ** *p* < 0.05; Only significant associations are shown; reference categories for OR: gender (male), age (13–15 years old), sleeping (≥8 h), using mobile phone (no use)

**Table 5:** Odds ratios (OR) and 95% confidence intervals (Cl) for multivariate associations with pain in last 7 days in the lower limbs

		Hips		Knees		Ankles/Feet	
		Uni	Multi	Uni	Multi	Uni	Multi
		OR; IC95%	OR; IC95%	OR; IC95%	OR; IC95%	OR; IC95%	OR; IC95%
Gender	Female	1.64; [1.06;2.53]**	1.61; [1.01;2.57]**	1.90; [1.34;2.69]**	1.99; [1.36;2.93]**	1.41; [0.98;2.03]*	1.55; [1.04;2.32]**
Sleeping hours	≤7 h	1.55; [0.99;2.43]*	1.36; [0.84;2.21]	1.79; [1.25;2.58]**	1.76; [1.20;2.58]**	1.90; [1.30;2.78]**	1.88; [1.26;2.80]**
Physical activity	Moderate	1.06;[1.01;1.11]**	1.06;[1.01;1.11]**	1.04;[1.01;1.09]**	1.03;[0.99;1.08]	1.08;[1.03;1.13]**	1.06;[1.01;1.11]**
	Vigorous	1.03;[0.99;1.09]		1.07;[1.03;1.11]**	1.08;[1.03;1.13]**	1.05;[1.01;1.10]**	1.05;[1.01;1.10]**
Using mobile phones	≤1 h	0.82;[0.38;1.74]	0.79;[0.36;1.74]	1.01;[0.55;1.84]	0.89;[0.48;1.67]	0.91;[0.50;1.66]	0.84;[0.45;1.58]
	[2;3] hours	1.02;[0.46;2.27]	0.91;[0.39;2.10]	1.12;[0.59;2.13]	0.87;[0.44;1.70]	0.85;[0.44;1.64]	0.73;[0.36;1.46]
	[4;5] hours	1.67;[0.68;4.08]	1.42;[0.55;3.64]	1.84;[0.88;3.84]	1.26;[0.58;2.74]	1.09;[0.50;2.39]	0.80;[0.34;1.84]
	≥5 h	2.56;[1.15;5.72]**	1.97;[0.83;4.66]	2.69;[1.38;5.24]**	1.75;[0.86;3.56]	1.96;[0.99;3.86]*	1.38;[0.66;2.88]

*p < 0.1; ** *p* < 0.05; Only significant associations are shown; reference categories for OR: gender (male), age (13–15 years old), sleeping (≥8 h), using mobile phone (no use)

### Pain and physical activity (univariable analysis)

Data on percentage of participants reporting moderate and vigorous PA and self-reported time spent in these activities is presented in Table 1. More time per week spent in moderate PA was significantly associated with increased probability of reporting pain in the last 7 days for all body sites except the mid back (percentage increases vary 4 and 9%, *p* < 0.05). More time per week spent in vigorous PA was significantly associated with increased probability of reporting pain in the last 7 days for the mid back, the shoulders, the knees and the ankles/feet (percentage increases vary between 4 and 7%) (Tables 3, 4 and 5).

### Pain and screen time (univariable analysis)

Data on self-reported screen time is presented in Table 1. Watching TV/DVDs and playing were not significantly associated with pain for any of the body sites considered. Using mobile phones for 5 h or more was significantly associated with increased odds of pain in the last 7-days for the mid back (OR = 2.74, *p* < 0.05), the low back (OR = 3.20, *p* < 0.05), the wrists (OR = 2.56, *p* < 0.05), the hips (OR = 2.56, *p* < 0.05) and the knees (OR = 2.69, *p* < 0.05). Using computers (all time intervals) was associated with increased odds of reporting pain in the low back (OR between 2.33 and 3.02, *p* < 0.05) (Table 3).

### Pain and sleep (univariable analysis)

Data on sleeping time is presented in Table 1. Reporting 7 h or less of sleep was significantly associated with increased odds of reporting pain in the last 7-days for the neck (OR = 1.98, *p* < 0.05), the mid back (OR = 2.16, *p* < 0.05), the lower back (OR = 1.64, *p* < 0.05), the wrists (OR = 1.87, *p* < 0.05), the knees (OR = 1.79, *p* < 0.05) and ankles/feet (OR = 1.90, *p* < 0.05) (Table 4).

### Pain in the last 7-days and gender, age, BMI, PA, screen time and sleep (multivariable binary logistic regression analysis)

In the multivariable models (Tables 3, 4 and 5), a significant association was found between more time spent in moderate PA and pain in the neck, the shoulders, the wrists, the hips and the ankles/feet (percentage increases between 6 and 8%, *p* < 0.05) and between time spent in vigorous PA and pain in the mid back, the knees and the ankles/feet (percentage increases between 5 and 8%, *p* < 0.05). Regarding screen time, a significant association was found between using computers up to 1 h (OR = 2.34, *p* < 0.05) and 2 to 3 h (OR = 2.43, *p* < 0.05) and pain in the low back. Sleeping 7 h or less was significantly associated with pain in the neck (OR = 2.05, *p* < 0.05), the mid back (OR = 2.11, *p* < 0.05), the wrists (OR = 1.79, *p* < 0.05), the knees (OR = 1.76, *p* < 0.05) and the ankles/feet (OR = 1.88, *p* < 0.05).

### Pain intensity in the last 7-days and demographic variables, BMI, number of pain sites, PA, screen time and sleep (multivariable linear regression analysis)

Neck pain intensity for the last 7-days was significantly associated with increased BMI (Coef. = 0.07;95% CI = [0.01;1.14]; Rp^2^ = 0.03), being a female (Coef = 0.70;95% CI = [0.17;1.24]; Rp^2^ = 0.04), using the computer for 4 h or more (Coef = 1.00;95% CI = [0.36;1.65]; Rp^2^ = 0.03) and using the mobile phone for 4 to 5 h (Coef = 0.86;95% CI = [0.22;1.50]; Rp^2^ = 0.04). Mid back pain intensity was significantly associated with being 16 years old or more (Coef = −1.23; 95% CI = [−1.87;-0.58]; Rp^2^ = 0.08), using the mobile phone for 5 h or more (Coef = 1.15; 95%CI = [0.40;1.90]; Rp^2^ = 0.03) and using the computer for 4 h or more (Coef = 0.79; 95% CI = [0.02;1.55]; Rp^2^ = 0.03). Low back pain intensity was significantly associated with number of pain sites (Coef = 0.28; 95% CI = [0.08;0.49]; Rp^2^ = 0.04) and using the mobile phone between 4 and 5 h (Coef = −0.94; 95% CI = [−1.54;-0.35]; Rp^2^ = 0.05).

Shoulder pain intensity was significantly associated with number of pain sites Coef = 0.29; 95% CI = [0.13;0.46]; Rp^2^ = 0.06) and using a mobile phone 2 to 3 h Coef = 0.81; 95% CI = [0.07;1.54]; Rp^2^ = 0.02) and ≥5 h (Coef = 1.32; 95% CI = [0.69;1.95]; Rp^2^ = 0.08).

**Fig. 1:**
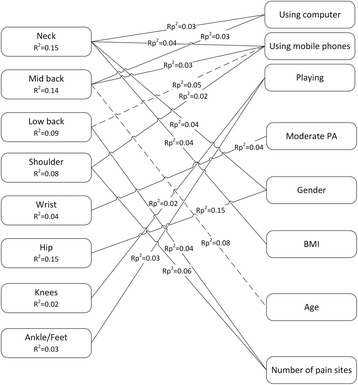
Schematic representation of the multivariable linear regression analysis for pain intensity (dependent variable) at the 8 different body sites. Rp2 = partial r squared; R2 = total r squared explained by the model; −--- negative significant association; ___ positive significant association

Hip pain intensity was significantly associated with being a female (Coef = 1.40; 95% CI = [0.77;2.03]; Rp^2^ = 0.15) and knee and ankle/feet pain intensity were significantly associated with playing for 4 h or more (Knee: Coef = 0.93; 95% CI = [0.19;1.67]; R^2^ = 0.02; Ankle/feet: Coef = 0.91; 95% CI = [0.11;1.71]; Rp^2^ = 0.03). Results of the multivariable analysis are schematically presented in Fig. 1.

### Pain associated disability in the last 7 days and demographic variables, BMI, number of pain sites, PA, screen time and sleep (linear regression analysis)

Of the 652 students, 371 (56.9) reported difficulty due to pain with at least one activity (Table 1). Pain was reported to disturb physical exercise classes (*n* = 182, 49.1%), leisure activities (*n* = 108, 29.1%), sitting during lessons (*n* = 102, 27.5%), sleep (*n* = 88, 23.7%) and walking more than 1 km (*n* = 22.6, 8.7%). Pain disability was significantly associated with being a female (Coef = 0.16; 95% CI = [0.03;0.30]; R^2^ = 0.01), number of pain sites (Coef = 0.15; 95% CI = [0.09;0.20]; R^2^ = 0.04) and mean pain intensity (Coef = 0.21; 95% CI = [0.17;0.25]; R^2^ = 0.18).

## Discussion

### Pain presence

This study explored the association between time spent sleeping, time spent in PA, time spent in screen based activities and the presence of pain at 8 different body sites, both in univariable and multivariable models. Results suggest that sleeping 7 h or less and spending more time in moderate PA are associated with increased odds of reporting pain at the neck and wrists; sleeping 7 h or less and spending more time in vigorous PA are associated with increased odds of reporting pain at the mid back and knee, while sleeping 7 h or less and spending more time in both moderate and vigorous PA are associated with pain in the ankle. Using computers for 1 h or more is associated with low back pain and spending more time in moderate PA is associated with increased odds of reporting both pain in the shoulder and hip. These results only partly support our hypothesis that the association between pain and PA, screen time and sleep will vary depending on the painful body site considered as results show similarities between the factors associated with pain (for example, both time spent sleeping and time spent in physical activity remained in the multivariate model for five body sites), but also show differences in relation to this pattern such as for pain in the low back, the shoulder and the hip. Similarly, some of the univariable significant associations became nonsignificant in the multivariable models, particularly for screen time, suggesting that physical activity and screen time may act as confounders on the association between screen time and pain. Taken together these findings seem to highlight the importance of considering sleeping, PA and screen time when studying associations with pain.

When comparing the findings of the present study with our own findings for predictors of chronic pain using the same independent variables in the models [23], there are similarities in the pattern of association for both pain in the last 7 days and in the last 3 months (Table 6). A possible explanation may be that we did not distinguish acute pain from chronic pain and for some students pain felt in the last 7 days could have been chronic pain, i.e., pain felt for 3 months or longer that was also present in the week before data collection. This also helps explain the high pain prevalence found in the present study. Nevertheless, it is also conceivable that pain, independently of its duration, shares common predictors. Future studies exploring factors associated with pain should distinguish between acute and chronic pain.

**Table 6:** Comparison of predictors of pain in the last 7-days and chronic pain (pain in the last 3 months or longer felt at least once a week)

Body site	Sex		Age		BMI		Sleep		Mod PA		Vig PA		TV/DVD		Playing		Phones		Computers	
	7D	3 M	7D	3 M	7D	3 M	7D	3 M	7D	3 M	7D	3 M	7D	3 M	7D	3 M	7D	3 M	7D	3 M
Neck	x	x		0			x	x	x	x								0		
Mid back	x	0		x			x	x		x	x					0	0	0		0
Low back	x	x		x			0	x	0	x		x					0		x	0
Shoulder	x	x	x	x				x	x	x	0	x						0		
Elbow										0	x	x								
Wrist	x	x		0			x	0	x	x		0						0		
Hip	x	x		x				0	x	x		0					0	0		0
Knees	x	x		x			x	0	x	x	x	x						0		
Ankle	x			0		0	x	0	x	x	x	x						0		0

*Mod* Moderate, *Vig* Vigorous, *PA* Physical activity, *0* Significant in the univariable model but not in the multivariable model, *x* Significant in the multivariable model

More time spent in moderate PA was associated with a 6 to 8% increased probability of reporting pain for 5 out of the 8 body sites investigated (neck, shoulders, wrists, hips and ankles/feet) and vigorous PA was associated with a 5 to 8% increased probability of reporting pain at 3 body sites (mid back, knees and ankles/feet). With the exception of the feet/ankle the significant association is either for one or for the other type of PA, suggesting that the operational definition of PA may influence study results and offering a possible explanation for the conflicting results of existing studies [5–7]. The association between pain and PA could be attributed to trauma, particularly in the lower limb [10]. However, this finding should not prevent adolescents from practicing PA as it has innumerous health benefits [13, 14]. Future studies should explore the mechanisms through which PA is associated with pain so that preventive strategies could be implemented.

Sleeping 7 h or less is associated with approximately two to threefold increased probability of reporting pain at 5 body sites (neck, mid back, wrists, knees and ankles/feet). There is evidence that sleep deprivation affect some fundamental mechanisms of pain and pain inhibition, which conceivably, have a global and not a local effect, such as dysregulation of the endogenous opioid system, increased negative mood in the presence of pain and increased pain catastrophizing [24, 25]. Despite consensus regarding an association between pain and sleep [26], previous studies investigating the association between musculoskeletal pain and sleep have considered musculoskeletal pain without discriminating body sites [27, 28] or have studied the association between sleep and a limited number of painful body sites [29]. A longitudinal study that investigated the ability of insufficient sleep to predict pain in the shoulder, the neck and the low back, concluded that insufficient sleep was a risk factor for NP and LBP but only in girls [29].

Using computers for 1 h or less and for 2 to 3 h was associated with a twofold increased probability (OR = 2.38 and 2.43, respectively) of reporting low back pain, suggesting that this is an important factor to consider for adolescents with low back pain. No other significant association was found in the multivariable model, suggesting, as already referred, a possible confounding effect of PA and/or sleep on the association between screen time and pain for the midback, the writs, the hips and the knees, where univariable associations were significant. An inverse association between screen-related discomfort and exposure to PA has been previously shown [30] as well as a mediating effect of sleep on the association between computer use and health symptoms [31]. Existing studies examining the association between screen time and pain show conflicting results and comparison with the findings of the present study is difficult as they tend to study pain in the back or upper limbs only and use chronic pain and univariable models (not accounting for confounding effects) [6, 8, 32]. Nevertheless, and in line with the present study findings, Briggs et al. [32] in a cross-sectional study using data from a cohort of 924 adolescents found no association between neck and shoulder pain and screen based activities. In contrast, Hakala et al. [8] found an increased risk of both low back (OR between 1.7 and 2) and neck pain (OR between 1.3 and 2.5) when using computers. Similarly to our study findings, Hakala et al. [8] found no association between neck and low back pain and time spent watching television and using mobile phones.

### Pain intensity

Our results suggest the factors associated with pain presence differ from those associated with pain intensity. Time spent in screen based activities (using the mobile phone, using computer and playing) emerged as the variable more consistently associated with pain intensity (all body sites except the wrist and the hip). In contrast, PA and time spent sleeping, which were associated with the presence of pain were not associated with pain intensity (except moderate PA for pain intensity at the wrist). Reasons for this discrepancy between the predictors of pain presence and predictors of pain intensity are outside the scope of this work. Nevertheless, several reasons may explain the association between time spent in screen based activities and pain intensity: i) the flexed and end of range postures that students tend to use during screen based activities [33, 34] may place excessive strain and/or stretch on sensitive structures; the long periods of time spent in static positions may further contribute to increase strain while also decreasing the appropriate oxygenation and removal of metabolites and algic substances from tissues, increasing nociceptive activity. There is some evidence in support of the proposed hypothesis: a prospective study on the association between posture during desk top computer use and pain found that increased head flexion predicted pain of higher intensity even when adjusted for psychosocial factors [35]; 1 h of combined workstation tasks resulted in decreased oxygen saturation and blood flow in all three parts of the trapezius muscle and 90 min of computer based work significantly increase pain intensity [36]. Nevertheless, these arguments do not seem to apply to low back pain as more time using mobile phones was associated with lower pain intensity. This is an unexpected association that needs to be investigated in future studies.

The percentage of pain intensity variance explained by the significant variables is low, suggesting that important predictors of pain intensity were not included. Psychological variables, such as anxiety, depression, catastrophizing or fear of movement have been shown to be associated with pain intensity [37, 38]. Whether the significant associations found in our model would remain in the presence of other relevant predictors needs to be explored in future studies.

### Disability

This study findings, similarly to previous studies, show that disability due to pain is highly prevalent in students. Hoftun et al. [2] in a sample of 7373 adolescents aged 13–18 years found that 79.6% of those with chronic pain reported difficulty in at least one daily activity. Being a female, having a higher number of pain sites and higher mean pain intensity was significantly associated with disability, but the amount of variance explained by the model is low. As for pain intensity, this may be related to the absence of any psychological variable in the model, as variables such as anxiety and pain catastrophizing are believed to be the most important predictors of pain associated disability for adolescents with pain [38–40]. Nevertheless, Hoftun et al.[2] showed that girls tend to report higher disability than boys and that scores in the disability index tend to increase with the number of pain sites (67.7% of those reporting pain in 5 body sites or more reported a disability index between 3 and 5 against 21.6% of those reporting only 1 body site). In a case-control study with 42 participants with musculoskeletal pain and 42 participants without pain aged 13 to 21 years old, both pain intensity and depressive symptoms predicted pain disability and no association was found between PA and pain disability [41].

### Study limitations and future work

The cross-sectional nature of this study did not allow for inferences on causality. The size of the sample was insufficient to perform subgroup analysis, considering for example the type of activity/sport practiced within moderate and vigorous physical activity or the number of painful body sites (e.g., neck pain only versus neck pain and pain at other body sites). In particular, pain at multiple body sites is usually associated with higher disability than single body site pain in adults [42]. Therefore, not to have included number of pain sites as a covariate when exploring which variables were associated with the presence of pain was also a limitation. The questionnaire did not differentiate acute pain felt in the last 7 days from non-acute pain (e.g., chronic pain) or pain of traumatic origin from idiopathic pain, neither discriminated disability associated with pain at different body sites. Participants are from a specific region what could compromise external validity. Nevertheless, the high response rate and the use of procedures that are similar to previous studies favour external validity. The study also involved multiple analyses, which had an exploratory nature. Nevertheless, it provides interesting results that need to be further explored in future studies.

## Conclusions

In summary, this study results suggest both similarities and differences in the patterns of association between time spent in PA, sleeping and screen based activities and pain presence at 8 different body sites. In addition, they also suggest that the factors associated with the presence of pain, pain intensity and pain associated disability are different.

## Abbreviations

BMI: Body mass index
NPQ: Nordic Musculoskeletal Questionnaire
PA: Physical activity
Rp2: Partial r square
